# Supplementary material for: Ecophysiological Differentiation among Two Resurrection Ferns and Their Allopolyploid Derivative
Source: Plants (Basel). 2023 Apr 1;12(7):1529. doi: 10.3390/plants12071529 (PMC10096763; doi:10.3390/plants12071529)
Supplement: Supplementary file 1 [file plants-12-01529-s001.zip › Supplementary materials_Tables and figures_FINAL.doc.pdf]

plants

MDPI

SUPPLEMENTARY MATERIALS

Data S1. Raw data in xlsx format.

Table S1. ANOVAs for testing differences in RWC and F_v/F_m among the three *Oesporangium* species after three desiccation levels (80%, 50%, and 10% relative humidities). $F_v/F_{m(desic.)}$ is the ratio (F_v/F_m after desiccation) : (initial F_v/F_m). Significant differences ($p < 0.05$) are indicated in bold. n = 17 individuals per species.

Variable	Source of variation	df [1]	SS	*F*	*p*
$RWC_{desic.}$	Species	2	8.6	0.07	0.9284
	Desiccation	2	3370.2	29.27	**<0.0001**
	Sp. × Desic.	4	76.7	0.33	0.8540
	Residual	41	2360.5		
$F_v/F_{m(desic.)}$	Species	2	1095.3	8.05	**0.0011**
	Desiccation	2	974.9	7.17	**0.0021**
	Sp. × Desic.	4	699.0	2.57	0.0521
	Residual	41	2788.3		

[1] df = degrees of freedom; SS = sum of squares

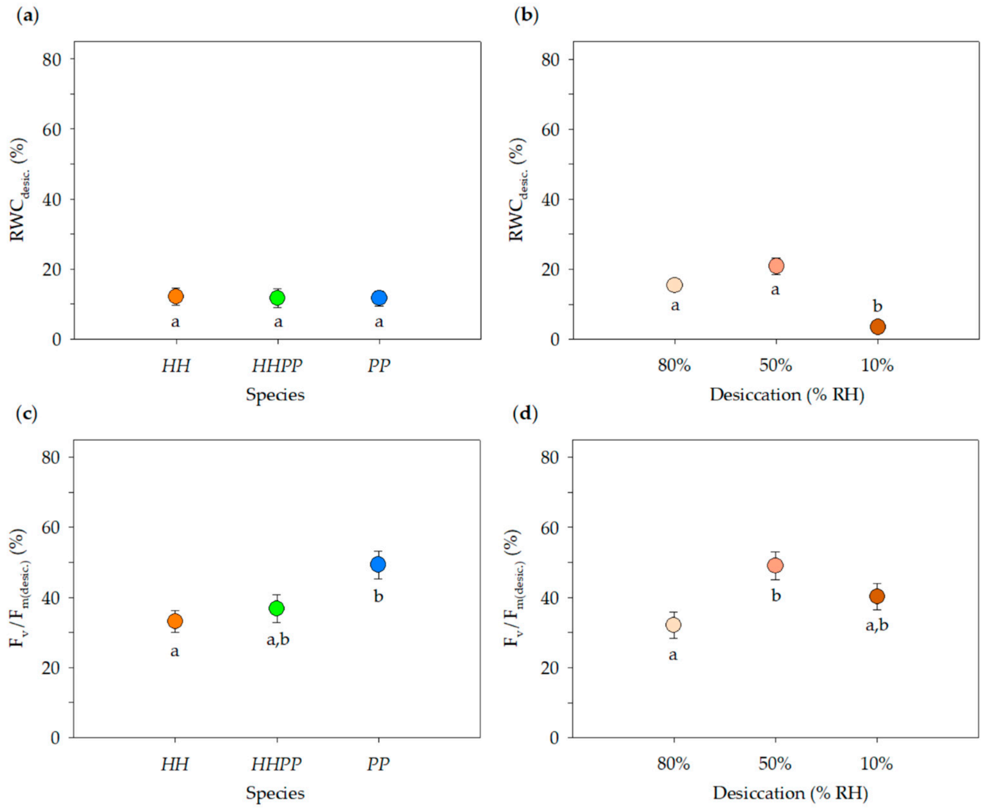

Figure S1. Mean values (± SE) of $RWC_{desic.}$ and $F_v/F_{m(desic.)}$ for the three *Oesporangium* species after three desiccation levels (80%, 50%, and 10% relative humidities): (**a**) $RWC_{desic.}$, comparison of species; (**b**) $RWC_{desic.}$, comparison of desiccation levels; (**c**) $F_v/F_{m(desic.)}$, comparison of species; (**d**) $F_v/F_{m(desic.)}$, comparison of desiccation levels. Different letters indicate significantly different means ($p < 0.05$, Tukey tests). n = 17 individuals per species. See Table S1 for ANOVA results.

Table S2. Mean values (± SE) of foliar elemental contents for the three *Oesporangium* species: aluminium (Al, mg kg^{-1}), calcium (Ca, g 100 g^{-1}), cadmium (Cd, mg kg^{-1}), iron (Fe, mg kg^{-1}), potassium (K, g 100 g^{-1}), magnesium (Mg, g 100g^{-1}), manganese (Mn, mg kg^{-1}), molybdenum (Mo, mg kg^{-1}), nickel (Ni, mg kg^{-1}), phosporus (P, g 100 g^{-1}), rubidium (Rb, mg kg^{-1}), sulfur (S, g 100 g^{-1}) and zinc (Zn, mg kg^{-1}). p values correspond to the comparison of species in one-way ANOVA; significant differences ($p < 0.05$) are indicated in bold. Different letters indicate significantly different means ($p < 0.05$, Tukey tests). These 13 elements were selected based on their variation explained by the PCA (Figure 4). n = 6–7 individuals per species. See Data S1 for other quantified elements.

		Species		
**Element**	***p***	***HH***	***HHPP***	***PP***
**Al**	**0.0002**	158.4 ± 16.4 a	343.4 ± 714 a	629.6 ± 83.6 b
**Ca**	**< 0.0001**	0.13 ± 0.02 a	0.23 ± 0.02 b	0.41 ± 0.03 c
**Cd**	**0.0044**	0.25 ± 0.03 a	0.20 ± 0.04 a,b	0.09 ± 0.02 b
**Fe**	**0.0021**	115.0 ± 9.7 a	186.6 ± 55.9 a	416.5 ± 36.1 b
**K**	**< 0.0001**	0.88 ± 0.06 a	1.22 ± 0.16 a	1.83 ± 0.09 b
**Mg**	**0.0014**	0.11 ± 0.01 a	0.18 ± 0.02 b	0.19 ± 0.01 b
**Mn**	**0.0034**	169.1 ± 28.3 a	150.2 ± 24.5 a	57.3 ± 2.0 b
**Mo**	**< 0.0001**	0.27 ± 0.02 a	0.44 ± 0.05 b	0.59 ± 0.03 c
**Ni**	0.4857	2.1 ± 0.3 a	2.8 ± 0.6 a	2.4 ± 0.4 a
**P**	**0.0009**	0.125 ± 0.004 a	0.158 ± 0.005 b	0.170 ± 0.010 b
**Rb**	**0.0053**	11.8 ± 1.2 a,b	16.0 ± 2.3 a	7.5 ± 1.1 b
**S**	**< 0.0001**	0.118 ± 0.004 a	0.142 ± 0.005 b	0.176 ± 0.005 c
**Zn**	0.1121	40.4 ± 7.0 a	54.7 ± 6.7 a	34.0 ± 6.9 a
